# Measurement of neutron energy spectrum at the radial channel No. 4 of the Dalat reactor

**DOI:** 10.1186/s40064-016-2585-7

**Published:** 2016-06-24

**Authors:** Pham Ngoc Son, Vuong Huu Tan

**Affiliations:** Nuclear Research Institute, 01-Nguyen Tu Luc, Dalat, Vietnam; Vietnam Agency for Radiation and Nuclear Safety, 113-Tran Duy Hung, Ha Noi, Vietnam

**Keywords:** Neutron spectrum, Neutron flux, Iterative adjusted spectrum

## Abstract

**Introduction:**

Several compositions of neutron filters have been installed at the channel No. 4 of the Dalat research reactor to produce quasi-monoenergetic neutron beams. However, this neutron facility has been proposed to enhance the quality of the experimental instruments, and to characterize the neutron spectrum parameters for new filtered neutron beams of 2 keV, 24 keV, 59 keV and 133 keV.

**Case description:**

In order to meet the demand of neutron spectrum information for calculation and design of filtered neutron facilities at the Dalat nuclear research reactor (DNRR), the experimental determinations of neutron flux and energy spectrum, up to 8 MeV, has been performed at the inner entrance of the horizontal channel No. 4 from the core of DNRR. The Westcott neutron fluxes as well as the α-parameter that represents the deviation of epithermal neutron distribution from the 1/E law were measured by applying the cadmium ratio and the multi-foils activation methods. The fast neutron spectrum was measured based on the iterative adjustment procedure with threshold reactions.

**Discussion and evaluation:**

A set of pure metal thin foils with the diameter of 1.27 cm and thickness of 0.125 mm were used as threshold detectors to measure the integrated fluxes, and a calculation procedure on iterative adjustment was implemented to derive the differential neutron energy spectrum from the integrated values.

**Conclusions:**

The neutron fluxes and spectrum parameters were characterized with the measured values of 4.80 × 10^9^, 1.98 × 10^7^, 5.06 × 10^8^ cm^−2^ s^−1^ and 0.0448 for the thermal, epithermal, fast neutron fluxes and the α-shape factor, respectively. The present result has been significantly applied to the input data for the Monte Carlo simulations in the developments of filtered mono-energetic neutron beam facility at the institute.

## Introduction and background

The Dalat research reactor was originally a TRIGA MARK II reactor with a nominal power of 250 kW completed construction and reached the critical state in 1963. The reactor then has been upgraded to the nominal power of 500 kW since 1984. There are three radial and one tangential neutron beam ports at DRR, each of which penetrates the concrete shield structure and the reactor water to provide external beams of neutron originated from the reactor core. The horizontal channel No. 4 is one of the radial channels of the Dalat nuclear research reactor. Several compositions of neutron filters have been installed in the channel to produce quasi-monoenergetic neutron beams of 0.0253, 54 and 148 keV, with neutron fluxes of 1.7 × 10^6^, 6.7 × 10^5^ and 3.9 × 10^6^ cm^−2^ s^−1^, respectively. The filtered neutron beams have been applied for measurement of neutron capture cross section, and for development of a prompt gamma neutron activation analysis system (PGNAA) in recent years. However, this neutron facility has been proposed to be renovated in order to enhance the quality of the experimental instruments, and to characterize the neutron beams parameters for calibrations of neutron detectors and dosimeters. This work is also required for expanding the channel for new filtered neutron beams of 2, 24, 59 and 133 keV. In this progress, it is requested to measure precisely the neutron energy spectrum, at the inner beam port position of the radial channel No. 4, for optimal simulation and design of the neutron filter configurations and the radiation shielding structure. This work presents the experimental procedures and measured results as a case study of differential neutron energy spectrum determination at the radial channel No. 4 of the Dalat research reactor.

### Conventional Westcott flux

In the Westcott’s conventions for thermal and epithermal neutron flux measurement (Westcott et al. 1958), the reaction rate per atom is expressed as:

1 $$R = nv_{0} \sigma_{0} \left[ {gG_{th} + r\sqrt {T/T_{0} } s_{0} G_{epi} } \right]$$

2 $$s = \frac{2}{\sqrt \pi }\frac{{I_{0}^{\prime } }}{{\sigma_{0} }}\sqrt {T/T_{0} } ;\quad s_{0} = s\sqrt {T_{0} /T}$$

where *G*_*th*_ and *G*_*epi*_ are the thermal and epithermal self-shielding factors, respectively. n*v*_0_ the neutron flux with the thermal neutron density and velocity (*v*_0_ = 2200 m s^−1^); σ_0_ is the cross section of neutron at 2200 m s^−1^. The Westcott factors (denoted as g and s) are functions of the temperature, which measure the departure of the cross section from the 1/*v*-law in the thermal and epithermal region; the temperature constant T_0_ is 293.6 K; s_0_ is the invariant quantity of s; $I_{0}^{\prime }$ is the reduced resonance integral which is subtracted by the 1/v part from the resonance integral I_0_ and the epithermal index r(T/T_0_)^1/2^. Therefore, the thermal neutron flux and the epithermal index can be determined by using a number of activation foils with different resonance characteristics as the following expression.

3 $$\frac{R}{{\sigma_{0} gG_{th} }} = nv_{0} + nv_{0} r\sqrt {T/T_{0} } \frac{{s_{0} G_{epi} }}{{gG_{th} }}$$

The Eq. (3) is a 1st order linear equation of intercept nv_0_ and slope nv_0_r(T/T_0_)^1/2^. If two different flux monitors are irradiated under the same condition, a common intercept and slope can be determined from the resulting reaction rates.

### Determination of α-factor in the E^−(1+α)^ epithermal neutron spectrum

In a thermal reactor, the flux distribution of the epithermal neutrons per unit energy interval is considered to be inversely proportional to the neutron energy. However, this assumption is valid only within an ideal moderating environment. In practices, this condition is rarely satisfied in a nuclear reactor. Therefore, the deviations from the 1/E law can occur in irradiation channels, and correction parameter (α) should be added into the resonance integral formula (Ryves 1969).

4 $$I = \int\limits_{{E_{Cd} }}^{\infty } {\frac{\sigma (E)}{E}dE}$$

where E_Cd_ is the Cadmium cut off energy.

In order to take into account all the above mentioned effects, the 1/E^1+α^ distribution (Ryves 1969; De Corte et al. 1981) has been used. Where α is an energy-independent coefficient, but it is depended on the environment of the neutron source facility. Accordingly, the modified formula for resonance integral is written as:

5 $$I(\alpha ) = \int\limits_{{E_{Cd} }}^{\infty } {\frac{\sigma (E)}{{E^{1 + \alpha } }}dE}$$

The relationship between I and I(α) is expressed as follows (De Corte et al. 1981).

6 $$I(\alpha ) = (I - 0.429\sigma_{0} )\overline{{E_{r} }}^{ - \alpha } + \frac{{0.429\sigma_{0} }}{2\alpha + 1}E_{Cd}^{ - \alpha }$$

where $\overline{{E_{r} }}$ is the effective resonance energy, considered as a nuclear constant, and $\sigma_{0}$ is the 2200 m s^−1^ neutron capture cross section. A method was developed by De Corte et al. (1981) for instantaneous α determination based on co-irradiation of three suitable resonance monitors as follows. The specific count rate for a given γ-peak, emitted from the irradiated sample, is defined as:

7 $$A_{sp} = \frac{1}{m}\frac{{N_{p} \lambda }}{{\left( {1 - e^{{ - \lambda t_{1} }} } \right)\left( {e^{{ - \lambda t_{2} }} } \right)\left( {1 - e^{{ - \lambda t_{3} }} } \right)}}$$

where t_1_, t_2_, t_3_ are the irradiation time, decay time, measurement time, respectively; N_p_ is the count number of the γ-peak; and m is the weight of irradiated sample. The specific count rate, A_sp_, can be also calculated within the Hogdahl’s convention (Høgdahl 1962) as the following expression:

8 $$A_{sp} = [f + Q(\alpha )]\phi_{epi} \sigma_{0} \varepsilon \gamma \theta C/M$$

where M, θ, γ and ε are atomic weight, isotope abundance, absolute intensity, and the detection efficiency of the γ-ray detector, respectively. Q(α) is the ratio of the resonance integral in the 1/E^(1+α)^ epithermal neutron spectrum to the (n, γ) thermal neutrons capture cross section σ_0_; and f is the ratio of thermal to epithermal fluxes.

9 $$\begin{aligned} & Q(\alpha ) = \frac{I(\alpha )}{{\sigma_{0} }} = \left( {Q - 0.429} \right)\overline{{E_{r} }}^{ - \alpha } + \frac{0.429}{2\alpha + 1}E_{Cd}^{ - \alpha } ; \\ & {\text{Q}} = {\text{ I}}/\sigma_{0} \\ \end{aligned}$$

The flux ratio, f, can be determined by using the co-irradiation of two suitable standard monitors, denoted as 1 and 2, according to the following equation (De Corte et al. 1981).

10 $$\begin{aligned} & f = \left( {\frac{{\phi_{th} }}{{\phi_{epi} }}} \right)_{1,2} = \left[ {\frac{{k_{1} \varepsilon_{1} }}{{k_{2} \varepsilon_{2} }}Q_{1} (\alpha ) - \frac{{A_{sp,1} }}{{A_{sp,2} }}Q_{2} (\alpha )} \right]\left[ {\frac{{A_{sp,1} }}{{A_{sp,2} }} - \frac{{k_{1} \varepsilon_{1} }}{{k_{2} \varepsilon_{2} }}} \right]^{ - 1} ; \\ & {\text{k}} =\upgamma \upsigma _{0}\uptheta/{\text{M}} \\ \end{aligned}$$

When using three resonance monitors, denoted as 1, 2 and 3, under the same irradiating conditions, the Eq. (10) can be written for each group 1–2 and 1–3. Making equality between the quantities f_1,2_ and f_1,3_ leads to the following expression:

11 $$F(\alpha ) = (a - b)Q_{1} (\alpha ) - (a + 1)Q_{2} (\alpha ) + (b + 1)Q_{3} (\alpha ) = 0$$

where

12 $$\begin{aligned} & a = \left[ {(A_{sp,1} k_{2} \varepsilon_{2} )/(A_{sp,2} k_{1} \varepsilon_{1} ) - 1} \right]^{ - 1} \\ & b = \left[ {(A_{sp,1} k_{3} \varepsilon_{3} )/(A_{sp,3} k_{1} \varepsilon_{1} ) - 1} \right]^{ - 1} \\ \end{aligned}$$

The coefficient α would be determined by solving the Eqs. (9), (11) and (12) with the experimental data of specific count rates. Once the coefficient α is obtained, the thermal and epithermal neutron spectrum can be expressed as the following formula (Beckurts and Wirtz 1964, p. 324):

13 $$\phi (E) = \phi_{th} E\frac{{e^{ - E/kT} }}{{(kT)^{2} }} + \phi_{epi} \frac{1}{{E^{1 + \alpha } }}\Delta (E/kT)$$

where *Δ*(*E*/*kT*) is the joined function, T temperature, and k the Boltzmann constant.

### Spectrum modification by interactive adjustments

The threshold reactions such as (n, p), (n, α), (n, 2n) and (n, n′) occur only if the neutron energy is above a particular threshold energy. In many cases, these reactions lead to radioactive products and can be used to measure the neutron flux density or energy spectrum in the fast energy region. In such a measurement, the threshold monitors should have cross sections as well as a step-function, which is zero below the threshold energy E_t_ and equal to constant σ above E_t_. Accordingly, the reaction rate can be written as:

14 $$R = \sigma \int\limits_{{E_{t} }}^{\infty } {\phi (E)dE}$$

But in practice, the real cross section is not an ideal step-function, and the approximation to the real cross sections is usually applied. The effective cross section and effective threshold energy are defined in such a way that the true reaction rate is obtained as follows (Beckurts and Wirtz 1964, p. 287).

15 $$\int\limits_{0}^{\infty } {\phi (E)\sigma (E)dE = \sigma_{eff} \int\limits_{{E_{eff} }}^{\infty } {\phi (E)dE} }$$

If a set of suitable threshold monitors, usually in the form of thin foils with different E_eff_ in the interest energy region, is irradiated under the same conditions, the neutron energy spectrum can be determined by the method of interactive adjustments. The least square procedure for spectrum modification by iterative adjustments is that the following expression be minimized as similar defines in Matzke (1994, p. 10):

16 $$S = \sum\limits_{i = 1}^{n} {\left[ {\ln \,A_{i} - \ln \sum\limits_{j = 1}^{m} {\sigma_{ij} \phi_{j}^{k} } } \right]^{2} + \sum\limits_{i = 1}^{n} {w_{ij} \sum\limits_{j = 1}^{m} {\left[ {\ln \,\phi_{j}^{k} - \ln \,\phi_{j}^{k - 1} } \right]^{2} } } }$$

where i, j, A_i_, k, and w denote the monitor, energy group, reaction rate of ith monitor, iterative step and weighting factor, respectively. The modified spectrum at the iteration step (k + 1) proceeds as follows:

17 $$\begin{aligned} & \phi_{j}^{k + 1} = \phi_{j}^{k} \exp \left( {M_{j}^{k} } \right) \\ & M_{j}^{k} = \frac{{\sum\nolimits_{i = 1}^{n} {w_{ij}^{k} \ln \left( {A_{i} /A_{i}^{k} } \right)} }}{{\sum\nolimits_{i = 1}^{n} {w_{ij}^{k} } }} \\ \end{aligned}$$

where w_ij_ is the weighting factor, which is approximation proportional to the relative response for ith detector weighting with the variances of the measured reaction rate for the monitor and the relevant cross section:

18 $$w_{ij}^{k} = \frac{{A_{ij}^{k} }}{{A_{i}^{k} }}\left( {\frac{1}{{\text{var} (A_{i} )}} \times \frac{1}{{\text{var} (\sigma_{ij} )}}} \right)$$

where $$A_{ij}^{k} = \phi_{j}^{k} \overline{{\sigma_{ij} }}$$ and $$A_{i}^{k} = \sum\nolimits_{j = 1}^{m} {A_{ij}^{k} .}$$

And $\overline{{\sigma_{ij} }}$ is the reaction cross section for ith detector, averaged constant over the jth energy interval. The iterative procedure is repeated until a solution is obtained due to the criteria. If it is realized in the n iteration step then the solution spectrum can be written as:

19 $$\phi_{j}^{n} = \phi_{j}^{0} \exp \left( {\sum\limits_{p = 0}^{n - 1} {M_{j}^{p} } } \right)$$

where $\phi_{j}^{0}$ represents the initial (input) spectrum which can be approximated by the fission spectrum of ^235^U.

## Case study

The above-mentioned methods have been applied to measure the partial neutron energy spectra corresponding to the energy ranges of thermal, epithermal and fast neutrons. The experiments were performed at inner entrance position of the horizontal channel ‘No. 4’ of the Dalat research reactor. The partial spectra were matched to each other to obtain a full energy spectrum.

### Thermal and epithermal neutron energy spectrum

#### Conventional Westcott fluxes

The activation monitors used for the determination of conventional Westcott fluxes were Co and Au, in disk form of thin foils with diameter of 1.27 cm (0.125 mm in thickness and 99.99 % purity). The irradiation times without Cd-cover were 5 min for Au and 30 min for Co. For irradiations with 0.7 mm Cd-cover, the irradiating times were 1 h for Au and 5 h for Co. The related nuclear data and parameters used in this experiment are given in Table 1 (Chadwick et al. 2006; Chatani 2003).

**Table 1:** Nuclear data and parameters of Au and Co monitors

Monitor	σ_0_ (barns)	*g*	$I_{0}^{\prime }$ (barns)	*s*_0_	*G*_*th*_	*G*_*epi*_
Au	98.70	1.005	1563.56	17.325	0.999	0.992
Co	37.18	1.000	74.0	1.782	0.999	0.997

The linear relation between the values of R/s_0_gG_th_ and s_0_G_epi_/gG_th_ in the Eq. (3) was obtained from the radioactive products of ^197^Au and ^59^Co monitors (without Cd cover). The thermal and epithermal self-shielding correction factors G_epi_ and G_th_ are from Chatani (2003). The results of thermal and epithermal neutron fluxes are obtained as ϕ_th_ = (4.80 ± 0.072) × 10^9^ cm^−2^ s^−1^ and ϕ_epi_ = (1.98 ± 0.031) × 10^7^ cm^−2^ s^−1^. The detail experimental values are shown in Table 2. The measured uncertainties are mainly estimated based on the statistical uncertainty of the gamma-peak area and the uncertainty of detection efficiency.

**Table 2:** Experimental results of epithermal index and Westcott fluxes

*nv*_0_ (cm^−2^ s^−1^) (%)	*nv*_0_ r(*T/T*_0_)^1/2^ (cm^−2^ s^−1^) (%)	*r*(*T/T*_0_)^1/2^ (%)
4.80 × 10^9^ ± 3.5	1.98 × 10^7^ ± 3.5	0.00413 ± 3.5

#### Determination of the α-parameter

With the experiments of *α*-determination, the pure thin foils of Au, In and Mn in diameter of 1.27 cm (0.125 mm in thickness and 99.99 % purity) were used as the three monitors. These monitors were irradiated under the same conditions for 10 min, and the specific activities of the samples after irradiation were measured by a calibrated HPGe detector with relative efficiency of 58 %. The nuclear data used in this experiment are given in Table 3 (Chadwick et al. 2006).

**Table 3:** Nuclear data of resonance monitors used for the *α*-parameter measurement

Monitor	reaction	M	θ (%)	σ_0_ (barn)	I_0_ (barn)	$\left\langle {{\text{E}}_{\text{r}} } \right\rangle$ (De Corte et al. 1981) (eV)	T_1/2_	E_γ_ (keV)	γ (%)
Au	^197^Au(n, γ)^198^Au	196.97	100	98.7	1563.56	5.47	2.697 d	411.8	95.53
In	^115^In(n, γ)^116m^In	114.82	95.7	164.3	2600	1.45	54.2 m	416.9	30.0
Mn	^55^Mn(n, γ)^56^Mn	54.98	100	13.4	11.76	337.1	2.582 h	847	99.99

The method proposed by De Corte et al. (1981) has been applied to determine the α-coefficient. The experiment has been performed at the same investigating site. The experimental value of α-coefficient is 0.0448 ± 0.0014, and the results on the intermediary parameters in Eq. (11) are also given in Table 4. The present results of neutron fluxes and α-parameter were introduced to the Eq. (13) in order to describe the energy-dependent spectrum of the thermal and epithermal neutrons, as shown in the Fig. 3.

**Table 4:** Measured values of α-coefficient, Q(α), a and b

α	*b*	*a*	*Q*(α)_*Au*_	*Q*(α)_*Mn*_	*Q*(α)_*In*_
0.045 ± 0.001	2.211 ± 0.055	−1.483 ± 0.037	14.54 ± 0.37	1.227 ± 0.031	16.48 ± 0.42

### Measurement of the fast neutron energy spectrum

**Fig. 1:**
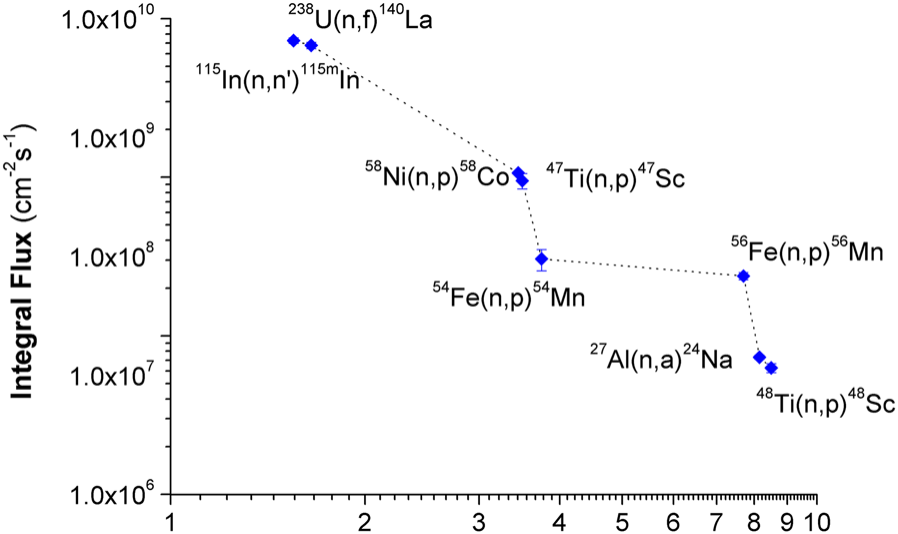
The integral fluxes measured with the threshold reactions

In practice, there are several methods for measurement of the fast neutron spectrum, but in the present experiment, the most suitable method is using the threshold reactions. Accordingly, six pure metal thin foils of In, U, Al, Fe, Ti, and Ni (0.125 mm in thickness and 99.99 % purity) were used as threshold monitors with various effective threshold energies, from 1.5 to 8.5 MeV. All the targets are in disk form with the same diameter of 1.27 cm. Each sample was covered by a cylindrical Cd-box, 0.7 mm in thickness, in order to reduce the influence of (n, γ) reaction with thermal neutrons. The irradiation times were 20 h for Fe and Al; 1 h for U and In; 4 h for Fe and Ni targets. The reaction rate of each monitor was calculated from the the activity measured by using a high resolution gamma-ray detector (HPGe, model GEM-50P4 Ortec). A computer procedure based on the iterative adjustment method (Zsolnay and Szondi 1982) was prepared to derive the differential energy spectrum from the experimental results of reaction rates. The effective energies, E_eff_, and effective cross sections, σ_eff_, were calculated from the evaluated data file ENDF/B-VI (Rose 1991). The experimental values of reaction rates per atom and the integral fluxes corresponding to the monitors are presented in the Table 5 and Fig. 1.

**Table 5:** The experimental results of integral fluxes and reaction rates

Reaction	E_eff_ (MeV)	Integral flux (cm^−2^ s^−1^)	σ_eff_ (barn)	Reaction rate/atom
^238^U(n, f)^140^La	1.55	6.56E+09	0.35	2.09E−15
^115^In(n, n′)^115m^In	1.65	5.98E+09	0.65	3.29E−16
^58^Ni(n, p)^58^Co	3.45	5.06E+08	0.6	3.04E−16
^47^Ti(n, p)^47^Sc	3.5	4.36E+08	0.13	5.66E−17
^54^Fe(n, p)^54^Mn	3.75	9.57E+07	0.4	3.83E−17
^56^Fe(n, p)^56^Mn	7.7	6.88E+07	0.13	8.95E−18
^27^Al(n, a)^24^Na	8.15	1.43E+07	0.12	1.72E−18
^48^Ti(n, p)^48^Sc	8.5	1.16E+07	0.065	7.57E−19

**Fig. 2:**
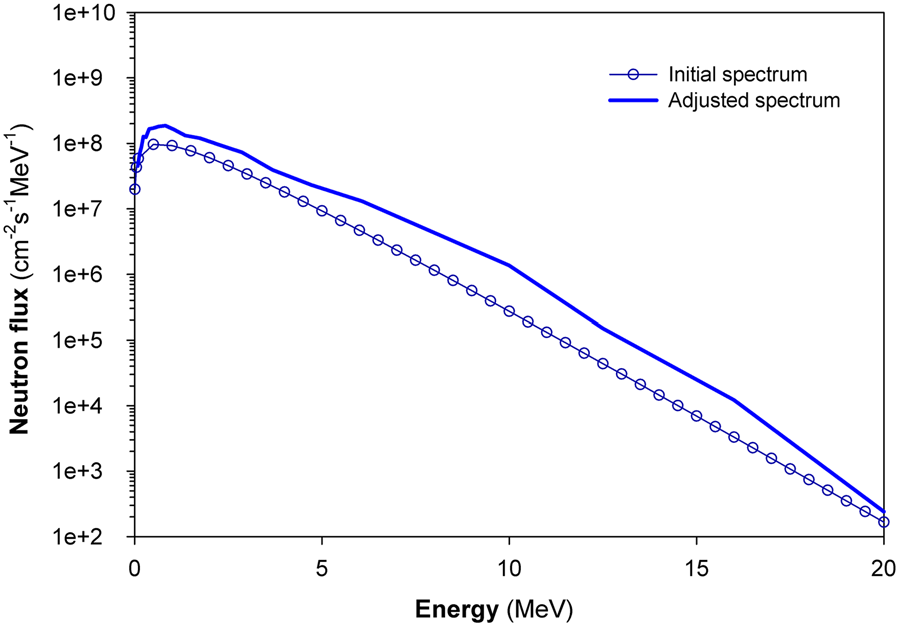
The adjusted spectrum for fast neutrons

**Fig. 3:**
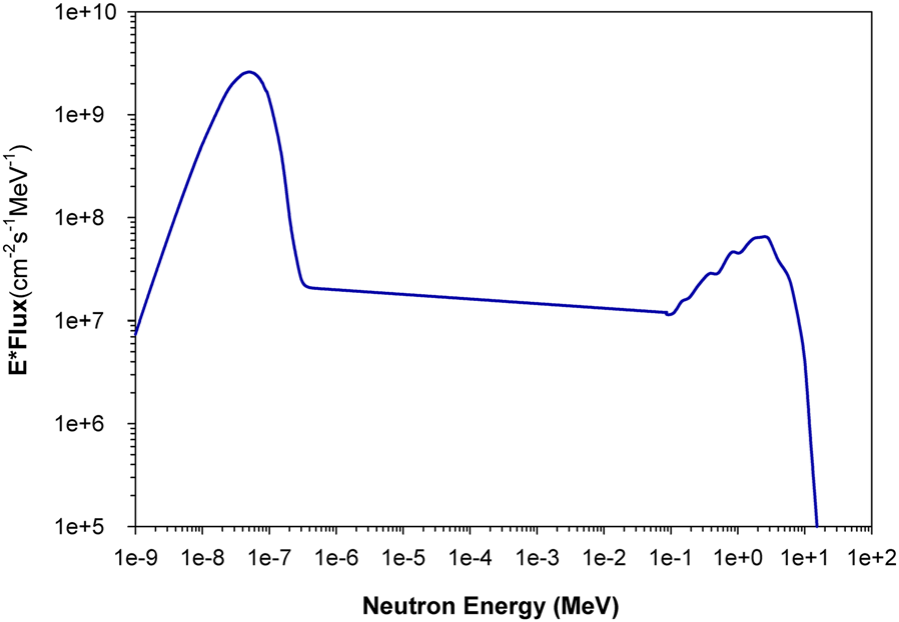
The result of differential neutron energy spectrum measured in this work

In the iterative adjustment process, the initial input spectrum, Φ_0_(E), was calculated from the empirical formula for unmoderated fission neutron spectrum produced from the fission of ^235^U as follows (William and Harry 1987, p. 145).

20 $$\varPhi_{0} (E) = \phi_{0} E^{1/2} \exp ( - E/E_{n} ) ,$$

where ϕ_0_ is the fast neutron flux which can be experimentally determined based on the ^58^Ni(n, p)^58^Co reaction, and E_n_ is the empirical constant that equal to 1.32 for fission neutron of ^235^U (William and Harry 1987, p. 145). The adjusted spectrum, as shown in the Fig. 2, is slightly higher than the initial spectrum. The reason of this difference is because the approximation value of ϕ_0_ was 2 × 10^8^ cm^−2^ s^−1^ were lower than the actual value of fast neutron flux that measured with the Ni monitor as presented in the Table 5. The full energy spectrum, including three parts of thermal, epithermal and fast neutrons has been obtained as shown in the Fig. 3.

## Conclusions

The differential neutron energy spectrum, at the inner entrance position of the radial channel No. 4 from the core of the Dalat nuclear research reactor, including the conventional Westcott fluxes, α-coefficient and fast neutron spectrum have been measured by the multi-foil activation method. The measured neutron energy spectrum show that, the thermal and epithermal neutron density fluxes are 4.80 × 10^9^ ± 0.072 and 1.98 × 10^7^ ± 0.031 cm^−2^ s^−1^, respectively. The experimental value of α-coefficient is 0.0448 ± 0.001. The energy neutron spectrum up to 10^7^ eV has been obtained. The present results are proposed to be used as input information for optimal simulations and design of neutron facilities and radiation shielding, which are under developing at the radial beam port No. 4 of the Dalat research reactor. We are expected to obtain collimated neutron beams, after transferring through a combination of filter materials, with a pure thermal flux higher than 10^6^ cm^−2^ s^−1^, or quasi-mono-energetic neutron flux of about 10^5^ cm^−2^ s^−1^ in keV energy region.

## References

[CR1] Beckurts KH, Wirtz K (1964). Neutron physics.

[CR2] Chadwick MB (2006). ENDF/B-VII.0 next generation evaluated nuclear data library for nuclear science and technology. Nucl Data Sheets.

[CR3] Chatani H (2003) Measurement of the Westcott conventionality thermal neutron flux and suchlike at irradiation facilities of the KUR. In: JAERI-conference 2003-006

[CR4] De Corte F, Hammami KS, Moens L, Simonits A, De Wispelaere A, Hoste J (1981). The accuracy of the experimental α-determination in the 1/(E^1 + α^) epithermal reactor neutron spectrum. J Radioanal Chem.

[CR5] Høgdahl OT (1962) Neutron absorption in pile neutron activation analysis. Report MMPP-226-1, The University of Michigan, Ann Arbor, Michigan

[CR6] Matzke M (1994) Unfolding of pulse height spectra: the HEPRO program system. Report PTB-N-19. Physikalisch-Technische-Bundesanstalt, Braunschweig

[CR7] Rose PF (ed) (1991) ENDF-201, ENDF/B-VI summary documentation. BNL-NCS-17541, 4th edn. National Nuclear Data Center, Brookhaven National Laboratory

[CR8] Ryves TB (1969). A new thermal neutron flux convention. Metrologia.

[CR9] Westcott CH, Walker WH, Alexander TK (1958) Effective cross sections and cadmium ratios for the neutron spectra of thermal reactors. In: Proceedings of the 2nd international conference in peaceful use of atomic energy, vol 16, A/C0NF.15/P/202, Geneva, pp 70–76

[CR10] William GC, Harry I, Kase KR, Bjarngard BE, Attix FH (1987). Neutron Spectroscopy. The dosimetry of ionizing radiation.

[CR11] Zsolnay EM, Szondi EJ (1982). Neutron spectrum determination by multiple foil activation method.

